# Editorial: Immunoregulation by adenosine signaling in infection and inflammation

**DOI:** 10.3389/fcell.2025.1586379

**Published:** 2025-03-13

**Authors:** Xiangsheng Huang, Weidong Zhao, Tingting Mills, Lei Shi, Xiaoyi Yuan

**Affiliations:** ^1^ Gastroenterology Research Center, Department of Internal Medicine, University of Texas Health Science Center at Houston, Houston, TX, United States; ^2^ Henan Key Laboratory of Immunology and Targeted Drugs, School of Medical Technology, Xinxiang Medical University, Xinxiang, China; ^3^ Department of Biochemistry and Molecular Biology, University of Texas Health Science Center at Houston, Houston, TX, United States; ^4^ RNA Oncology Group, School of Public Health, Lanzhou University, Lanzhou, China; ^5^ Department of Anesthesiology, Critical Care and Pain Medicine, University of Texas Health Science Center at Houston, Houston, TX, United States

**Keywords:** adenosine, adenosine receptor, immune regulation, inflammation, therapy

## Introduction

Inflammation is a critical physiological defense mechanism for maintaining homeostasis and responding to injury or infections, yet its dysregulation drives chronic pathologies, such as autoimmune disorders, lung inflammation and cardiovascular diseases. Adenosine, a purine nucleoside derived from ATP hydrolysis, plays a dual role in modulating immune responses, exhibiting both pro- and anti-inflammatory effects contingent on the cellular and environmental contexts. Under physiological conditions, adenosine levels are tightly regulated, but during metabolic stress, hypoxia, or tissue injury, extracellular adenosine concentrations rise via equilibrative nucleoside transporters (ENT1/ENT2), activating 4 G protein-coupled receptors (A1, A2A, A2B, A3) to mediate context-dependent immune responses. This Research Topic highlights adenosine’s multifaceted roles in inflammatory diseases and cancer therapies, emphasizing its therapeutic potential while addressing the challenges of balancing efficacy and safety.

## Adenosine’s role in immune modulation

Adenosine receptors mediate diverse effects in inflammation, immunity, and tissue repair. The A2A receptor is predominantly anti-inflammatory, suppressing pro-inflammatory cytokines (e.g., TNF-α, IL-1β), reducing neutrophil activation, and promoting regulatory T cell (Treg) expansion, thus maintaining immune tolerance. A2B receptors exhibit context-dependent effects: in cancer, they foster immunosuppression by supporting a tumor-promoting microenvironment, while in inflammatory bowel disease (IBD), they mitigate inflammation and enhance tissue repair by promoting IL-10 production and macrophage polarization. Additionally, A1/A2A receptor crosstalk mitigates neuroinflammation in Alzheimer’s disease, and inhaled adenosine analogs targeting these receptors show promise in reducing IL-17-driven inflammation. Collectively, adenosine balances pro- and anti-inflammatory effects across various diseases, highlighting its potential as a therapeutic target for autoimmune disorders, chronic inflammation, and cancer.

## Tissue-specific roles of adenosine

*Gut Inflammation*: Vuerich et al. highlighted recent breakthroughs in understanding the role of adenosine in IBD, including Crohn’s disease (CD) and ulcerative colitis (UC). The CD73/adenosine pathway is known for its critical role in regulating inflammation and immune responses by acting on different immune cells. Adenosine signaling via A2A and A3 receptors suppresses inflammation by inhibiting NF-κB signaling and promoting epithelial repair. Dysregulation of this pathway, such as reduced CD39^+^ Th17 cells in CD or downregulated A2A and A3 receptors in UC, exacerbates inflammation and tissue damage. Therapeutically, adenosine receptor agonists like ATL-146e and IB-MECA show promise in experimental models by restoring immune balance and enhancing tissue repair. These findings underscore the therapeutic potential of modulating purinergic signaling in combination with conventional treatments to manage chronic inflammatory diseases like IBD effectively.

*Radiation-induced lung injury (RILI)*, including pneumonitis and fibrosis, involves a complex interplay between pro-inflammatory and anti-inflammatory responses, with CD73/adenosine signaling playing a pivotal role. Gockeln et al. specifically summarized the interactions between radiotherapy and CD73/adensoine pathways in RILI. CD73 modulates inflammation by promoting macrophage polarization to an anti-inflammatory M2 phenotype and facilitating tissue repair. However, prolonged adenosine signaling can drive fibrosis via extracellular matrix deposition and sustained macrophage activation. In non-small cell lung cancer (NSCLC), CD73 overexpression fosters tumor immune escape by suppressing cytotoxic T cells and expanding Tregs, which correlates with poor prognosis. While targeting CD73 with therapies like Oleclumab enhances anti-tumor immunity in radio-immunotherapy by mitigating immunosuppression, it may exacerbate immune-related adverse events (irAEs), such as pneumonitis by prolonging ATP-driven inflammation in the absence of adenosine-mediated resolution. These findings underscore the need for precise modulation of CD73/adenosine dynamics to optimize cancer control while minimizing pulmonary toxicity in combined radio-immunotherapy regimens.

*Cardiovascular Diseases*: Liu et al. demonstrated the combination of digilanid C and electroacupuncture stimulation (ES) demonstrates significant potential in addressing cardiac lipid metabolism dysregulation and remodeling in chronic heart failure (CHF). This combination attenuate lipid accumulation and suppress aberrant cardiac glial cell (CGC) activation by activates the JAK1/STAT3 pathway, which accompanied by reduced IL-6 expression and glutamatergic signaling. Adenosine is known for its critical complementary role by modulating mitochondrial function, and neurovascular repair through its A2A and A3 receptors, which suppresses IL-6 production and inflammatory damage and enhances mitochondrial biogenesis (Csoka et al., 2007; Doyle et al., 2025). Additionally, adenosine’s ability to regulate neurovascular coupling aligns with the observed improvements from digilanid C-ES therapy. This integrated approach underscores the therapeutic potential of combining adenosine modulation with digilanid C-ES to target metabolic dysregulation, inflammation, and neurovascular dynamics in CHF.

## Therapeutic potential

MicroRNAs (miRNAs) and purinergic signaling exhibit a dynamic interplay in regulating inflammation and immune responses, offering promising therapeutic strategies. Zhou et al. highlights the critical role of microRNAs (miRNAs) as regulators of immune and inflammatory responses in diabetic nephropathy. miRNAs, such as miR-146a and miR-16 regulate adenosine receptor expression and downstream pathways, influencing macrophage polarization, cytokine suppression (e.g., IL-6, IL-1β), and T cell activity (Mastroianni et al., 2019; Tian et al., 2016). Conversely, adenosine signaling modulates miRNA expression, as demonstrated by methotrexate-induced adenosine release activating miR-181b to mitigate vascular inflammation (Sun et al., 2012). These bidirectional interactions highlight opportunities for combined miRNA-adenosine therapies to fine-tune immune balance in inflammatory diseases. In cancer therapy, anti-CD73 agents like Oleclumab enhance radio-immunotherapy efficacy by sustaining ATP-driven immune activation, as seen in the COAST trial, with Oleclumab and durvalumab. However, CD73 blockade risks exacerbating radiation pneumonitis due to unresolved ATP-driven NLRP3 inflammasome activation and IL-1β/IL-18 release, as observed in trials like PACIFIC. Balancing these effects requires precise modulation of ATP-adenosine dynamics to optimize anti-tumor immunity, underscoring the need for integrated strategies that harmonize miRNA regulation, adenosine signaling, and immune checkpoint modulation in therapeutic design.

## Challenges and future directions

The findings emphasize the importance of individualized therapeutic strategies for targeting adenosine signaling, particularly in cancer and inflammatory diseases. Precision medicine approaches can optimize the timing, dosage, and delivery of CD73-targeted therapies to enhance anti-tumor efficacy while minimizing adverse effects, such as pneumonitis. Future research should focus on delineating the context-dependent roles of adenosine signaling in acute versus chronic inflammation and the long-term consequences of its pharmacological modulation. Advancements in dual-targeting strategies combining adenosine modulation with other pathways (e.g., PD-1/PD-L1 blockade) could further enhance efficacy while tailoring interventions to specific disease contexts.
